# The Influence of Diagnoses of Specific Viral Infections on In-Hospital Mortality, Length of Stay and Cost in Patients Admitted to Hospital with a Diagnosis of Myocarditis: An Analysis of the National Inpatient Sample

**DOI:** 10.31083/j.rcm2407206

**Published:** 2023-07-17

**Authors:** Chun Shing Kwok, Maximilian Will, Deddo Moertl, Adnan I. Qureshi, Josip A. Borovac

**Affiliations:** ^1^Department of Post Qualifying Healthcare Practice, Birmingham City University, B15 3TN Birmingham, UK; ^2^Department of Cardiology, University Hospitals of North Midlands NHS Trust, ST4 6QG Stoke-on-Trent, UK; ^3^Department of Internal Medicine 3, University Hospital St. Pölten, Karl Landsteiner University of Health Sciences, 3500 Krems, Austria; ^4^Department of Neurology, Zeenat Qureshi Stroke Institute, University of Missouri, Columbia, MO 65212, USA; ^5^Division of Interventional Cardiology, Cardiovascular Diseases Department, University Hospital of Split, 21000 Split, Croatia

**Keywords:** myocarditis, viral infection, mortality, length of stay, cost

## Abstract

**Background:**

The influence of different viral infections in patients with myocarditis is unknown. Myocarditis is an inflammatory disease of heart muscle that is commonly caused by viruses. The impact of different viral infections in patients with myocarditis is unknown.

**Methods:**

We conducted a retrospective cohort study using data between 2016–2020 in the National Inpatient Sample in the USA to evaluate admissions with myocarditis and concomitant viral infection. The outcomes of in-hospital mortality, length of stay (LoS), and cost, among patients hospitalized for myocarditis was evaluated.

**Results:**

A total of 27,050 hospital admissions for myocarditis were included and 6750 (25.0%) had a co-diagnosis of viral infection. Patients with myocarditis and viral infection had significantly higher mortality compared to those without viral infection (23.6% *vs*. 4.4%, *p* < 0.001). Viral infection was associated with increased in-hospital mortality (odds ratio (OR) 2.03, 95% CI 1.51 to 2.73, *p* < 0.001), greater median LoS (7 *vs*. 3 days, *p* < 0.001) and median hospitalization cost ($21,445 *vs*. $11,596, *p* < 0.001), compared to patients without viral infection. The rate of death was greatest for patients with a diagnosis of coronavirus disease 2019 (COVID-19), viral pneumonia and herpes zoster, respiratory syncytial virus, chronic hepatitis, and influenza which was 36.0%, 34.3%, 27.3%, 21.4%, 20.0%, and 14.5%, respectively.

**Conclusions:**

In conclusion, the diagnosis of viral infection is present in one in four patients hospitalized with myocarditis and is correlated with greater mortality, LoS, and in-hospital cost.

## 1. Introduction

Myocarditis is an inflammatory disease of heart muscle that can be caused by the broad array of infectious and non-infectious conditions. Myocarditis is heterogeneous in terms of clinical presentation and severity and as such might range from asymptomatic state with self-limiting clinical course up to fulminant myocarditis with life-threatening consequences and severe complications such as cardiogenic shock or ventricular arrhythmias [1]. The diagnosis of myocarditis might also be challenging and should employ multimodality integrative diagnostic approach comprising of (and not limited to) biomarker evaluation, electrocardiography, transthoracic echocardiography, cardiovascular magnetic resonance (CMR) imaging, and endomyocardial biopsy (EMB) as the established method for the diagnosis of myocarditis [2]. Reliable estimates of incidence of myocarditis can be challenging to evaluate as patients may not present to healthcare professionals with mild illness, and clinicians do not investigate all patients with the suspected clinical diagnosis with imaging or endomyocardial biopsy. Nevertheless, the condition is important as it might be associated with prolonged hospital admissions and poor clinical outcomes. For example, 30% of EMB-confirmed myocarditis cases progress to dilated cardiomyopathy while some types of myocarditis such as giant-cell myocarditis carry a staggering 90% rate of death or transplantation [2, 3].

There are many common causes for myocarditis which can be classified as infectious etiologies such as viral, parasitic, bacterial, and fungal agents and noninfectious etiologies such as toxins, hypersensitivity reactions and immunological syndromes [3]. Viruses are the most frequent cause of acute myocarditis among infectious pathogens [4]. Knowledge about viral myocarditis is incomplete and there are no effective treatment options [5]. While many viruses have been implicated to cause myocarditis the most common include adenovirus and enteroviruses such as coxsackieviruses [6]. Parvovirus B19 has been linked to myocarditis and its progression towards dilated cardiomyopathy [7]. A review of influenza myocarditis suggests that it is a rare condition and complications are even rarer but fulminant myocarditis can be fatal [8]. Other viruses such as Human Immunodeficiency Virus (HIV) [9], herpes zoster virus (HZV) [10], herpes simplex virus (HSV) [11], and cytomegalovirus [12] have also been reported to be associated with myocarditis. A recent study evaluated the influence of the diagnosis of viral infections on in-hospital outcomes for patients with heart failure [13] but the same type of evaluation has not been investigated for myocarditis. In this study, we report on viral infection diagnoses in patients who are hospitalized with a principal discharge diagnosis of myocarditis. We compared the characteristics of patients with myocarditis who had a concomitant viral infection to patients with myocarditis but without registered viral infection with respect to endpoints such as in-hospital mortality, length of stay (LoS), and cost from a nationwide perspective.

## 2. Materials and Methods

This manuscript is written according to the guidance of the STrengthening the Reporting of OBservational studies in Epidemiology (STROBE) checklist [14]. Ethical approval was not required as we analyzed a non-identifiable dataset.

We analyzed data that is nationally representative of the United States in the National Inpatient Sample (NIS). The NIS is a dataset produced by the Healthcare Cost and Utilization Project (HCUP). It is the largest publicly available inpatient healthcare dataset in the United States that can be analyzed to evaluate national figures on inpatient healthcare utilization, access, costs, quality, and outcomes [15].

A retrospective cohort study was performed by analyzing hospital records in the United States with a diagnosis of myocarditis between 2016 to 2012. The years of data were selected because implantable cardioverter-defibrillator (ICD)-9 codes were used before 2016. The diagnosis of myocarditis or the purposes of the study was based on ICD-10 codes I40 and I41 and we do not have more detailed information about the use of cardiac magnetic resonance imaging to ascertain the diagnosis or whether it was a clinical diagnosis. We excluded patients with age less than 18 years, or those that had missing values for death, and sex.

We defined patients who had viral infections based on the individual diagnoses of viral infections based on ICD-10 diagnostic codes and procedure codes as outlined in **Supplementary Table 1**. These viral infections included coronavirus disease 2019 (COVID-19), influenza, viral pneumonia, viral gastroenteritis, viral meningitis/encephalitis, HSV, HZV, acute viral hepatitis, chronic viral hepatitis, HIV, cytomegalovirus, infectious mononucleosis, viral conjunctivitis, adenovirus, enterovirus, parvovirus, and respiratory syncytial virus. The discharge diagnosis codes were used to identify coexisting illnesses and demographic data, hospital data and outcome data (in-hospital mortality, and length of stay) were available in the NIS dataset. The procedural codes were used to identify the need for intubation. In-hospital mortality was the primary outcome, and hospital length of stay and cost were the secondary outcomes.

### Statistical Analysis

Statistical analysis was undertaken on Stata 13 (version 13, College Station, TX, USA). National estimates were obtained by taking the hospital admissions and weighting them by the discharge weight as recommended by HCUP [14]. The weighted sample was stratified by those which had any viral infection and no diagnosed viral infection. Descriptive statistics were determined with the percent for categorical variables and median and interquartile range (IQR) for continuous variables. The non-parametric equality-of-medians test on Stata and the ${\mathrm{Chi}}^{2}$ tests were used to determine if there were any statistical differences for continuous variables and categorical variables, respectively. The frequency of individual diagnoses of viral infections was determined and the rate of mortality for each diagnosis of viral infection. The median length of stay and median cost for the individual diagnoses were determined. Multiple logistic regressions were used to estimate the independent odds of in-hospital mortality with any compared to no viral infection diagnosis. Stratified adjustments were performed in several models: (a) no adjustments, (b) adjustments for age and sex, (c) adjustments for age, sex, demographics and hospital variables, (d) adjustments for age, sex, demographics, hospital variables, comorbidities, and endomyocardial biopsy, (e) adjustments for age, sex, demographics, hospital variables, comorbidities, endomyocardial biopsy, heart failure, acute myocardial infarction, pericarditis, and shock and, finally, a full model adjusted for age, sex, demographics, comorbidities, endomyocardial biopsy, heart failure, acute myocardial infarction, pericarditis, shock, sepsis, respiratory failure/arrest, intubation, and ventilation.

Demographic variables were defined by race, smoking status, alcohol misuse, elective admission, weekend admission, season, year, primary expected payer, zone improvement plan (ZIP) income quartile, and hospital bed size. Comorbidities included obesity, arterial hypertension, hypercholesterolemia, diabetes mellitus, previous myocardial infarction, atrial fibrillation, valvular heart disease, infective endocarditis, previous stroke, peripheral vascular disease, chronic kidney disease, liver failure, chronic lung disease, cancer, dementia, and immunodeficiency.

Additional analysis was performed to evaluate the co-existing viral infection diagnoses for patients with viral pneumonia. Adjusted multivariable linear regression models were utilized to define the impact of viral infection diagnosis on length of stay and cost.

## 3. Results

The flow diagram of patients admitted to hospital with myocarditis is shown in **Supplementary Fig. 1**. There were 27,050 weighted hospital admissions of patients with a myocarditis and 6750 had a co-diagnosis of viral infection.

The patient characteristics for the admissions with myocarditis stratified according to a diagnosis of any viral infection or no such diagnosis are shown in Table 1. Admissions with a diagnosis of viral infection were older (median 59 *vs.* 43 years, *p* < 0.001) and a greater proportion were female (41.8% *vs. *38.6%, *p* = 0.001). There were more admissions from patients of Black (20.5% *vs*. 16.6%) and Hispanic (18.2% *vs*. 12.6%) and fewer patients of white ethnicity (50.9% *vs*. 63.0%) among patients with a diagnosis of viral infection. There were a small proportion of admissions where the patients had private health insurance in the group with viral infections (34.1% *vs*. 50.0%).

**Table 1.** **Characteristics and comorbidities of patients with and without viral infections with a hospital diagnosis of myocarditis**.

Variable		Admission without a diagnosis of viral infection (n = 20,300)	Admission with a diagnosis of viral infection (n = 6750)	*p*-value
Median age in years [IQR]		43 [29 to 59]	59 [40 to 72]	<0.001
Female sex		38.6%	41.8%	0.035
Race				<0.001
	White	63.0%	50.9%	
	Black	16.6%	20.5%	
	Hispanic	12.6%	18.2%	
	Asian or Pacific Islander	3.3%	4.7%	
	Native American	0.7%	0.9%	
	Other	3.9%	4.7%	
Smoking		1.3%	0.5%	0.017
Alcohol misuse		2.3%	1.6%	0.10
Elective admission		3.7%	3.2%	0.38
Weekend admission		25.6%	28.7%	0.025
Season				<0.001
	Spring	25.9%	33.2%	
	Summer	22.5%	17.0%	
	Fall	23.9%	21.1%	
	Winter	27.7%	28.7%	
Year				<0.001
	2016	17.2%	6.5%	
	2017	20.2%	9.2%	
	2018	21.2%	11.3%	
	2019	20.7%	9.3%	
	2020	20.7%	63.8%	
Primary expected payer				<0.001
	Medicare	19.7%	38.9%	
	Medicaid	18.2%	17.2%	
	Private insurance	50.0%	34.1%	
	Self-pay	8.1%	5.7%	
	No charge	0.6%	0.3%	
	Other	3.4%	3.9%	
ZIP income quartile				0.011
	1st–25th	25.5%	29.1%	
	26th–50th	24.8%	26.1%	
	51st–75th	25.0%	23.1%	
	76th–100th	24.8%	21.7%	
Hospital bed size				0.067
	Small	15.8%	15.6%	
	Medium	25.9%	29.1%	
	Large	58.3%	55.3%	
Obesity		16.8%	19.4%	0.029
Systolic arterial hypertension		43.3%	57.1%	<0.001
Hypercholesterolemia		25.2%	32.6%	<0.001
Diabetes mellitus		15.6%	30.4%	<0.001
Previous myocardial infarction		4.4%	4.6%	0.81
Atrial fibrillation		12.8%	20.2%	<0.001
Valvular heart disease		7.8%	5.6%	0.007
Infective endocarditis		2.0%	1.0%	0.026
Previous stroke		3.4%	6.2%	<0.001
Peripheral vascular disease		1.7%	2.2%	0.31
Chronic kidney disease		10.4%	21.5%	<0.001
Liver failure		5.9%	9.2%	<0.001
Chronic lung disease		16.6%	19.6%	0.011
Cancer		5.2%	4.9%	0.66
Dementia		0.8%	6.4%	<0.001
Immunodeficiency		0.4%	1.1%	0.006
Endomyocardial biopsy		4.0%	1.9%	<0.001
Heart failure		43.1%	44.2%	0.49
Pericarditis		6.7%	3.5%	<0.001
Acute myocardial infarction		19.4%	30.2%	<0.001
Shock		14.0%	19.6%	<0.001
Sepsis		12.3%	40.2%	<0.001
Respiratory failure or arrest		20.8%	48.0%	<0.001
Dependence on ventilator		0.6%	2.9%	<0.001
Intubation		6.5%	22.7%	<0.001
In-hospital mortality		4.4%	23.6%	<0.001
Median length of stay [IQR]		3 [2 to 7]	7 [3 to 14]	<0.001
Median cost [IQR]		$11,596 [7430 to 22,106]	$21,445 [10,295 to 50,117]	<0.001

ZIP, zone improvement plan; IQR, interquartile range.

In terms of comorbidities, there were greater proportion of admissions with concomitant viral infection that had chronic kidney disease (21.5% *vs*. 10.4%, *p* < 0.001), liver failure (9.2% *vs*. 5.9%, *p* < 0.001), chronic lung disease (19.6% *vs*. 16.6%, *p* = 0.011), dementia (6.4% *vs*. 0.8%, *p* < 0.001), and immunodeficiency (1.1% *vs*. 0.4%, *p* = 0.006). Endomyocardial biopsy was performed in 4.0% of admissions without a diagnosis of viral infection and in 1.9% of admissions with a diagnosis of viral infection (*p* < 0.001). Among admissions with viral infections, there was a greater incidence of sepsis (40.2% *vs*. 12.3%, *p* < 0.001), acute myocardial infarction (30.2% *vs*. 19.4%, *p* < 0.001), and shock (19.6% *vs*. 14.0%, *p* < 0.001). More admissions with viral diagnosis had respiratory failure or arrest (48.0% *vs*. 20.8%, *p* < 0.001), dependence on ventilator (2.9% *vs*. 0.6%, *p* < 0.001), and intubation (22.7% *vs*. 6.5%, *p* < 0.001). The median length of stay was greater for admissions where the patient had a viral infection diagnosis (7 *vs*. 3 days, *p* < 0.001) and the cost was greater for those with a diagnosis of viral infection ($21,445 *vs*. $11,596, *p* < 0.001). The crude unadjusted mortality rate was more than double for admissions where a patient had diagnosis of a viral infection diagnosis compared to those without a diagnosis of viral infection (23.6% *vs*. 4.4%, *p* < 0.001).

**Fig. 1.**
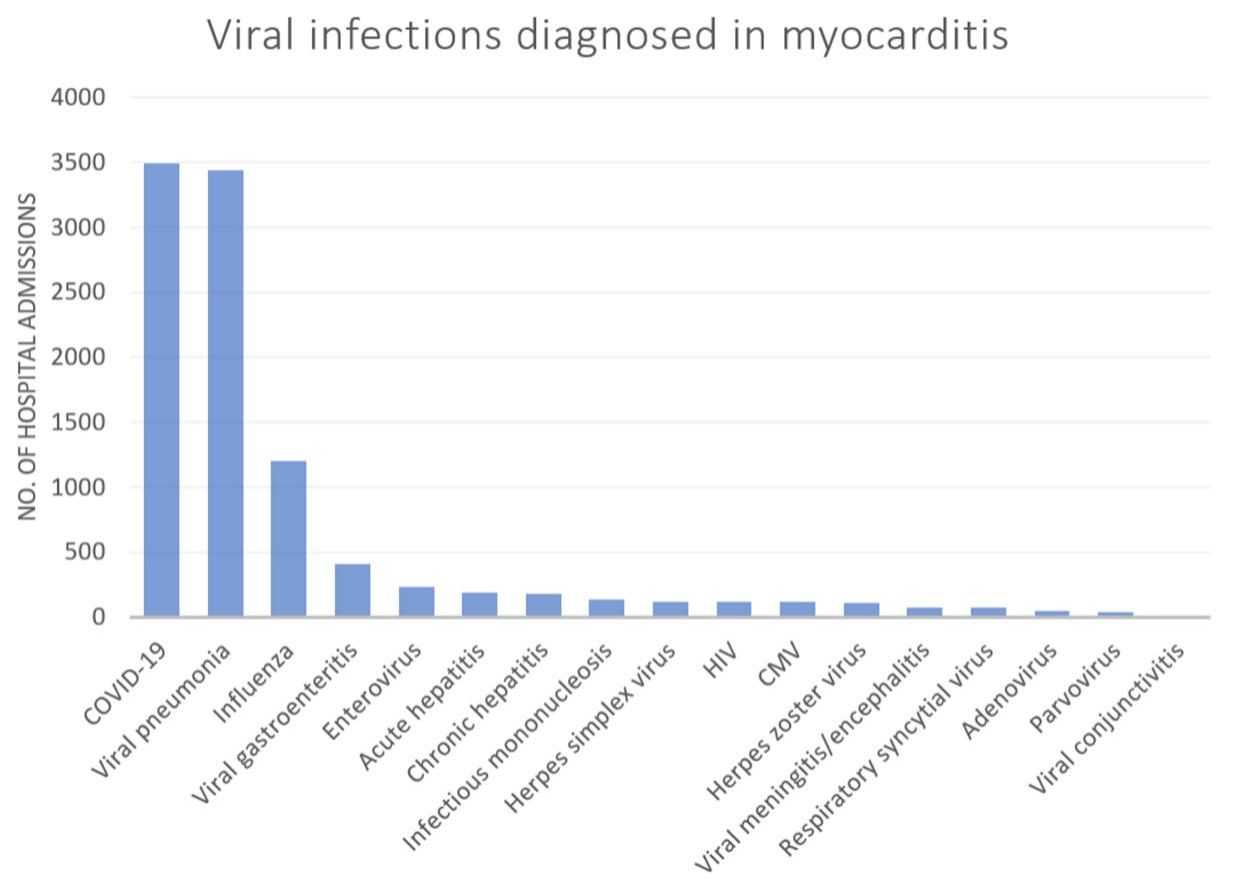
**Admissions for viral infections among patients who are admitted with a diagnosis of myocarditis**. CMV, cytomegalovirus; HIV, human immunodeficiency virus; COVID-19, coronavirus disease 2019.

The number of admissions with patients admitted to hospital with myocarditis stratified by the specific type of viral infections is shown in Fig. 1. There were 3495, 3440 and 1205 admissions with a diagnosis of COVID-19, viral pneumonia, and influenza as co-diagnosis in patients with myocarditis which represented the 3 most diagnosed viral infections in this group. The major diagnosis of viral infection among patients with viral pneumonia was COVID-19 (80.7%) (**Supplementary Table 2**).

**Fig. 2.**
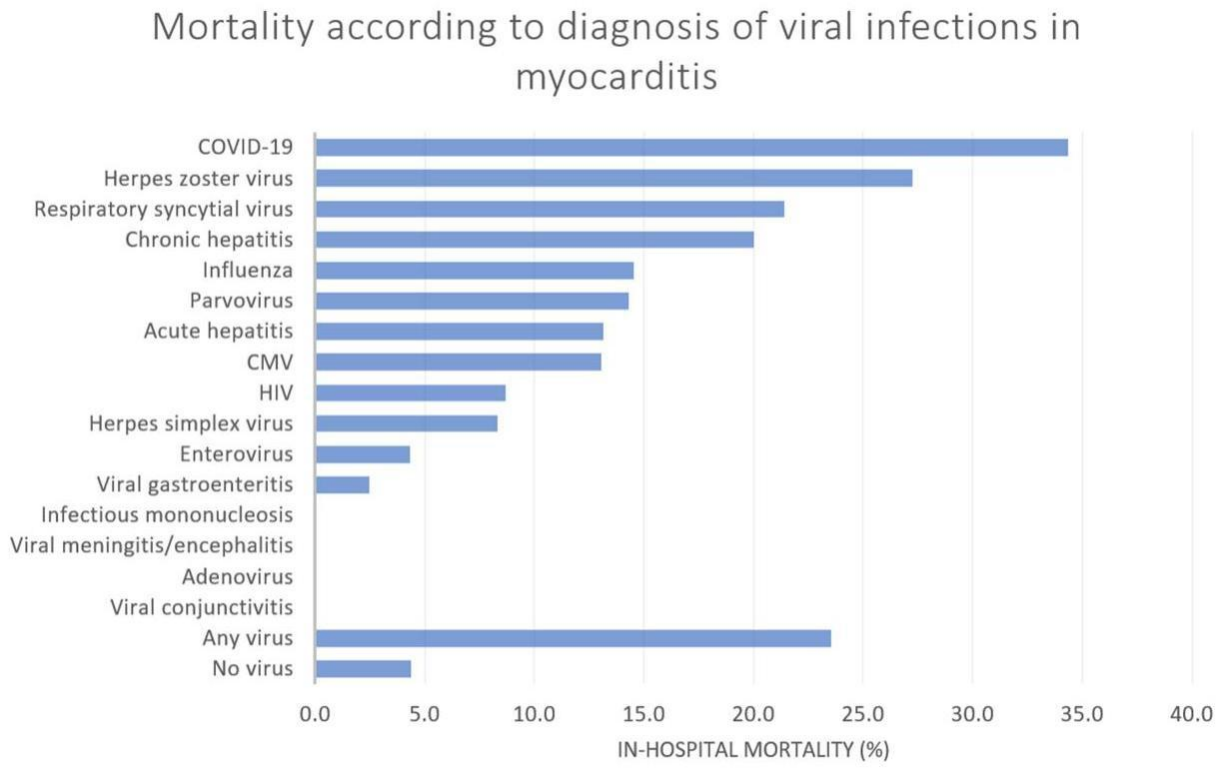
**In-hospital mortality rate for patients admitted with a diagnosis of myocarditis and viral infection**. CMV, cytomegalovirus; HIV, human immunodeficiency virus; COVID-19, coronavirus disease 2019.

The in-hospital mortality rate according to specific viral infections is shown in Fig. 2. The in-hospital mortality was greatest for admissions for patients with a diagnosis of COVID-19, herpes zoster, respiratory syncytial virus, chronic hepatitis, and influenza, which was 36.0%, 34.3%, 27.3%, 21.4%, 20.0 and 14.5%, respectively. No in-hospital deaths occurred in admissions with a diagnosis of infectious mononucleosis, viral meningitis/encephalitis, adenovirus, and viral conjunctivitis.

**Fig. 3.**
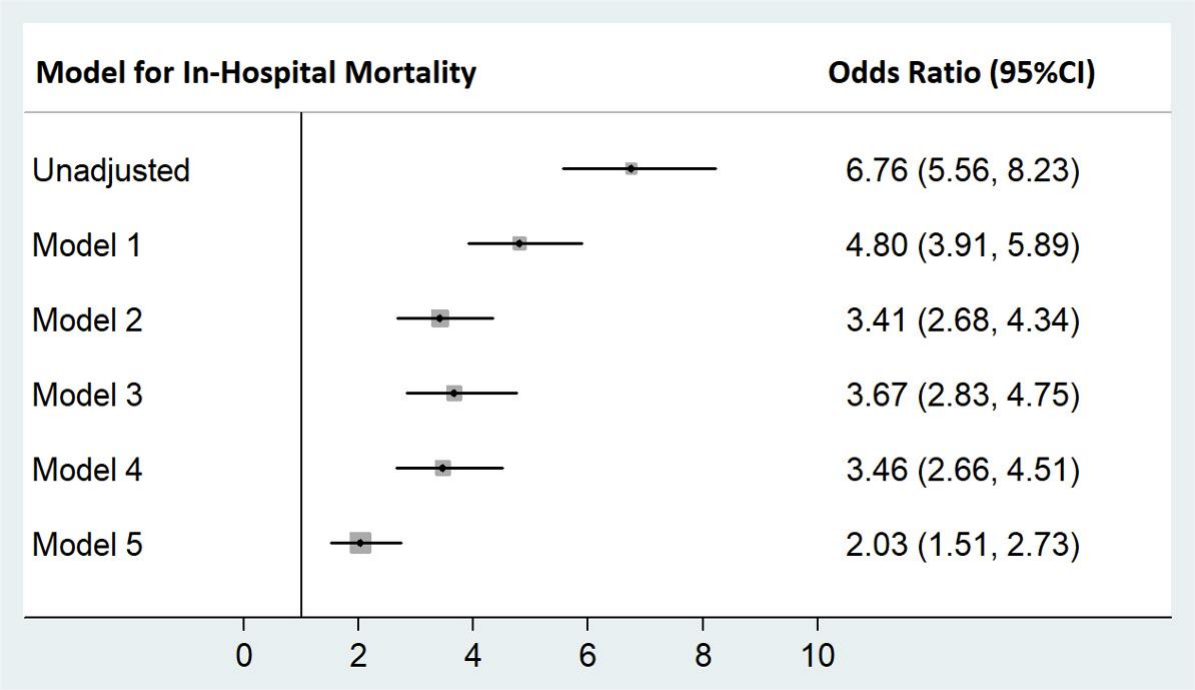
**Odds of in-hospital mortality for patients admitted with a diagnosis of myocarditis and any viral infection**. Model 1 adjusted for age and sex; Model 2 adjusted for Model 1 + demographics + hospital variables; Model 3 adjusted for Model 2 + comorbidites + endomyocardial biopsy; Model 4 adjusted for Model 3 + heart failure, acute myocardial infarction, pericarditis and shock; Model 5 adjusted for Model 4 + sepsis, respiratory failure/arrest, intubation, and mechanical ventilation.

The multivariable-adjusted odds of mortality of any viral infection diagnosis compared to no viral infection diagnosis is shown in Fig. 3. There was an unadjusted 6-fold increase in odds of in-hospital mortality for patients with a diagnosis of viral infection (odds ratio (OR) 6.76, 95% CI 5.56 to 8.23, *p* < 0.001). After adjustments for age, sex, demographics, comorbidities, endomyocardial biopsy, acute myocardial infarction, heart failure, shock, pericarditis, sepsis, intubation, ventilation and respiratory failure/arrest, there was a two-fold increase in odds of mortality associated with viral infection (OR 2.03, 95% CI 1.51 to 2.73, *p* < 0.001). The receipt of endomyocardial biopsy was not a significant predictor of in-hospital mortality (OR 1.18, 95% CI 0.62 to 2.26, *p* = 0.62).

Table 2 provides information regarding the length of stay and hospitalization cost for patients who are admitted with myocarditis with viral infections. The length of stay was greatest for those with HSV infection (median 24 days) and cytomegalovirus (median 17 days), and the median costs for admission were $90,449 and $67,407, respectively. After adjustments, any diagnosis of viral infection was associated with significantly increased length of stay (linear regression coefficient 1.42, 95% CI 0.58 to 2.26, *p* = 0.001) but there was no significant difference in cost (linear regression coefficient 3749, 95% CI –1542 to 9039, *p* = 0.17).

**Table 2.** **Median length of stay and median cost for patients with and without viral infections with a hospital diagnosis of myocarditis**.

Diagnosis	Median length of stay (days)	Median cost (USD)
Herpes simplex infection	24 [6 to 40]	$90,449 [21,129 to 208,302]
Cytomegalovirus	17 [8 to 35]	$67,407 [33,631 to 172,965]
Viral conjunctivitis	10 [2 to 13]	$22,801 [11,840 to 32,259]
Human immunodeficiency virus	9 [4 to 26]	$38,748 [15,775 to 114,562]
Viral pneumonia	9 [5 to 18]	$28,907 [14,197 to 61,750]
COVID-19	9 [4 to 17]	$26,020 [11,921 to 57,475]
Herpes zoster infection	7 [3 to 13]	$17,009 [12,632 to 46,440]
Chronic hepatitis	6 [3 to 13]	$23,502 [10,045 to 59,435]
Enterovirus	6 [2 to 12]	$22,433 [8831 to 36,286]
Respiratory syncytial virus	6 [2 to 8]	$20,557 [13,997 to 36,113]
Parvovirus	5 [4 to 13]	$16,095 [12,569 to 51,865]
Influenza	5 [3 to 11]	$16,666 [8990 to 44,071]
Viral meningitis/encephalitis	4 [2 to 12]	$24,038 [10,972 to 35,157]
Acute hepatitis	4 [3 to 10]	$11,619 [8654 to 26,971]
Infectious mononucleosis	4 [2 to 9]	$12,631 [8210 to 23,855]
Adenovirus	4 [3 to 7]	$18,481 [11,048 to 32,259]
Viral gastroenteritis	2 [1 to 4]	$9816 [5954 to 15,343]
Any viral infection	7 [3 to 14]	$21,445 [10,295 to 50,117]
No viral infection	3 [2 to 7]	$11,596 [7430 to 22,106]

COVID-19, coronavirus disease 2019.

## 4. Discussion

This large nationwide analysis of hospital admissions with a diagnosis of myocarditis provides several key findings. First, a quarter of hospital records where the patient was diagnosed with myocarditis have a co-diagnosis of viral infection and 3.5% of hospital records with a diagnosis of myocarditis had an endomyocardial biopsy. Second, the patients with a diagnosis of viral infections were more likely to be older and female and present with heart failure, myocardial infarction, shock, respiratory failure or arrest, dependence on ventilators, and intubation. Third, the most common specific viral infections were COVID-19, viral pneumonia, and influenza, and the in-hospital mortality rate was greatest for patients with a diagnosis of viral pneumonia, COVID-19, herpes zoster, respiratory syncytial virus, chronic hepatitis, and influenza. Finally, there was a two-fold increase in odds for mortality among patients with any diagnosis of viral infection compared to no diagnosis of viral infection and length of stay and cost were much higher for patients with diagnosed viral infection.

A key consideration of the current findings is that the vast majority patients with myocarditis did not have endomyocardial biopsy. Most cases of myocarditis are believed to be caused by a viral infection [16]. In order to confirm viral myocarditis, evaluation of cardiac tissue is required, and it has been reported that viral co-infections are only found in 12% of myocarditis cases [17]. The higher rate of viral co-infections reported in the current study may reflect the COVID-19 diagnoses that are not present in previous studies. It is not possible to know how the diagnosis of myocarditis was made and some patients could have had a clinical diagnosis of myocarditis without investigations. Furthermore, the diagnosis of acute viral infection may not be sought especially in cases where the acute phase of systemic infection was over and the presentation to hospital was for symptoms of myocarditis. The other consideration is that the population receiving endomyocardial biopsy may be different from those who do not receive biopsy. It is possible that the patients who receive endomyocardial biopsy may have worse clinical condition which merit tissue confirmation of whether the etiology was viral or not. This was supported by the observation that patients who had viral diagnoses had a greater proportion of patients with heart failure, shock, sepsis, respiratory failure or arrest, intubation, and ventilation. Also, the diagnosis of myocarditis is based on ICD-10 codes and there were codes corresponding to acute myocarditis (I40) and other myocarditis (I41). The vast majority (97.8%) of patients had the diagnosis of acute myocarditis. The small sample of other myocarditis (2.2%) was insufficient for more detailed multivariable analysis. Nevertheless, it is notable that the mortality rate for other myocarditis is more than double that of acute myocarditis (19.5% *vs*. 8.9%).

Our findings suggest that most patients with myocarditis in hospital do not have a secondary diagnosis of viral infection and those with specific viral infection diagnoses have high mortality, length of stay, and cost for hospitalization. There are a few national studies that evaluate myocarditis from the national perspective. Shah *et al*. [18] evaluated 27,129 hospitalizations with a primary diagnosis of myocarditis between 2007 and 2014 and found that more men were hospitalized compared to women (66% *vs*. 34%) while mortality was greater in women compared to men (3.5% *vs*. 1.8%, adjusted OR 1.69, 95% CI 1.1 to 2.6, *p* = 0.007). Elbadawi *et al*. [19] evaluated 22,299 hospital admissions with a diagnosis of myocarditis over 16 years (1998–2013) and found that 3.6% had endomyocardial biopsy and those who had biopsy had a two-fold increase in in-hospital mortality and greater stay in hospital. Another study focused on cardiogenic shock and use of mechanical circulatory devices in patients with myocarditis and found that in-hospital mortality was 4.4% and cardiogenic shock increased from 6.9% in 2005 to 12.0% in 2014 with a parallel increase in use of extracorporeal membrane oxygenation or percutaneous cardiopulmonary support and percutaneous ventricular assist devices [20]. Our current evaluation builds on the literature by evaluating a more contemporary cohort and we consider the different specific viral infections and their impact on in-hospital outcomes.

Our study suggests that COVID-19 had a major impact on patients with myocarditis. Events were only captured in the year 2020 but 41.1% of patients with myocarditis had this diagnosis. Whether directly from the impact of the infection on patients or indirectly through changes in the management of patients, mortality in 2020 was much higher at 18.6% compared to the average of 4.8% in the years before. The patients with COVID-19 had significantly greater death rate of 34.3% compared to 7.6% for patients without a diagnosis of COVID-19. These findings support the 3-fold increase in adjusted odds of mortality associated with myocarditis compared to no myocarditis in COVID-19 patients that has been recently reported in a propensity matched analysis [21].

The low incidence of myocarditis necessitates large scale data on the condition to identify co-diagnoses of viral infections. Even using nationally representative data from the United States, we found that over 4 years there were only 27,050 cases. As testing for specific viral infections are unlikely to have taken place for all patients with clinical diagnosis of myocarditis, and the test may also come back negative due to false negative testing, it is necessary to have such large sample in order to identify rare specific viral infections. In addition, patients may not be aware they have myocarditis and do not present to healthcare professionals. It may further be argued that for mild cases where the myocarditis is low risk, the testing for viral infection may not change clinical management so patients may not be tested.

The main question related to testing is whether early identification of the viral cause could have averted adverse outcomes. This is particularly important as 9.2% of patients die, and these patients that died also had co-diagnosis of heart failure and features of respiratory failure/arrest, need for intubation and ventilation and sepsis. While not all viral infections have treatments, it is possible that if testing took place earlier for patients, then more aggressive supportive therapy could be initiated as delay to escalation to intensive care can impact eventual outcomes.

The adult population evaluated with myocarditis in the United States merits discussion. The patients in this study are young and on average in their fourth decade of life who were in greater proportion male, and Caucasian. Young patients have fewer comorbid illnesses than older patients and have greater physiological reserve. The private health insurance is interesting because there may be differential care depending on the extent of healthcare coverage and the requirement of patients to part subsidize the care they receive. Also, pericarditis was not common in patients with myocarditis and was only present in 6% of patients and these patients with perimyocarditis had reduced mortality compared to myocarditis alone (4.4% *vs*. 9.4%). Interestingly, 22.1% of patients with myocarditis had a diagnosis of acute myocardial infarction. Both conditions share common features of elevated troponin, but acute myocardial infarction implies there is coronary artery disease. However, even with coronary disease, the scar pattern on cardiac magnetic imaging can help differentiate coronary disease from myocarditis. In addition, shock was also present in 15.4% of patients and it was more common in patients with a viral diagnosis. This suggests that some of the patients are hemodynamically unstable, and this raises the question of whether some infections may be more prone to developing shock, and whether patients could have presented earlier before they met the criteria for the clinically shocked state.

Our study found that patients with perimyocarditis had better prognoses compared to patients who have myocarditis alone. The good prognosis for pericarditis with and without myocardial involvement has been the conclusion of a multicenter prospective cohort study of 486 patients [22]. However, this study captured no mortality events in any of the groups after 36 months of follow up. The current evaluation of national data from the United States captured mortality events because of its large sample size. Future studies should investigate why patients with myopericarditis do better than those with myocarditis alone.

There are a few clinical implications for this work. Testing patients who may be at risk of deterioration for viral infections may be helpful as some viruses such as cytomegalovirus and herpes simplex virus may have antiviral medications. Also, as COVID-19 and influenza is the most diagnosed viral infection, this study may support the need for greater uptake of the COVID-19 and influenza vaccination program. In the United States, currently everyone 6 months and older should get an influenza vaccine every season [23] yet only 50% of adults are vaccinated from 2020 to 2021 [24]. The vaccination is perhaps an effective way of reducing the burden of the viral infection induced myocarditis as it would reduce hospitalization and mortality. However, it is important that the decision to be vaccinated is informed as Center for Disease Control and Prevention has reported that myocarditis and pericarditis have rarely been reported after the second dose of the vaccine [25]. A challenge for patients who have had COVID-19 infection and the vaccine prior to myocarditis is whether the either or both the vaccine or the infection contributed to the development of the myocarditis.

This evaluation has several limitations. First, we do not have data about the proportion of patients tested for viral infection and the mode of testing. We also do not know whether those who were diagnosed with viral infections had clinical diagnoses only or laboratory confirmed tests. Secondly, the study is of retrospective design and the data is observational, so it is subjected to potential confounding. In particular, we did not collect data on ventricular arrhythmias or complete heart block which are serious complications of myocarditis. Third, we do not have information about the management of patients including testing formation such as plasma troponin levels, left ventricular ejection fraction, imaging test findings, and histology report for diagnoses together with any treatments received other than intubation and dependence on the ventilator. Fourth, the NIS dataset does not enable identification of individuals so the same patients may appear more than once in the same year and across different years. Finally, this dataset included hospitalization until 2019, the pre-COVID-19 era, and thus we cannot extrapolate our findings to the COVID-19 pandemic.

## 5. Conclusions

In conclusion, one in four hospitalized patients with myocarditis have a secondary diagnosis of viral infection. The most common infections were COVID-19, viral pneumonia, and influenza. These patients have greater length of stay, cost, and in-hospital mortality. Future studies are needed to understand if more infections may be identified with greater testing, patient outcomes can be improved with earlier viral infection detection, and the burden of viral myocarditis from influenza and COVID-19 may be reduced with vaccination.

## Data Availability

The data used for this analysis may be purchased from the Healthcare Cost and Utilization Project website. The authors do not have permission to share the data used for the analysis.

## Abbreviations

CMR, cardiovascular magnetic resonance; EMB, endomyocardial biopsy; HIV, Human Immunodeficiency Virus; HZV, herpes zoster virus; HSV, herpes simplex virus; NIS, National Inpatient Sample; HCUP, Healthcare Cost and Utilization Project; IQR, interquartile range; OR, Odds ratio.

## Supplementary Material

Supplementary material associated with this article can be found, in the online version, at https://doi.org/10.31083/j.rcm2407206.
