# Supplementary material for: The Influence of Diagnoses of Specific Viral Infections on In-Hospital Mortality, Length of Stay and Cost in Patients Admitted to Hospital with a Diagnosis of Myocarditis: An Analysis of the National Inpatient Sample
Source: Rev Cardiovasc Med. 2023 Jul 17;24(7):206. doi: 10.31083/j.rcm2407206 (PMC11266461; doi:10.31083/j.rcm2407206)
Supplement: Supplementary file 1 [file 2153-8174-24-7-206-s1.docx]

**Supplementary Fig. 1. Flow diagram of hospital admissions with myocarditis included in the analysis.**

**
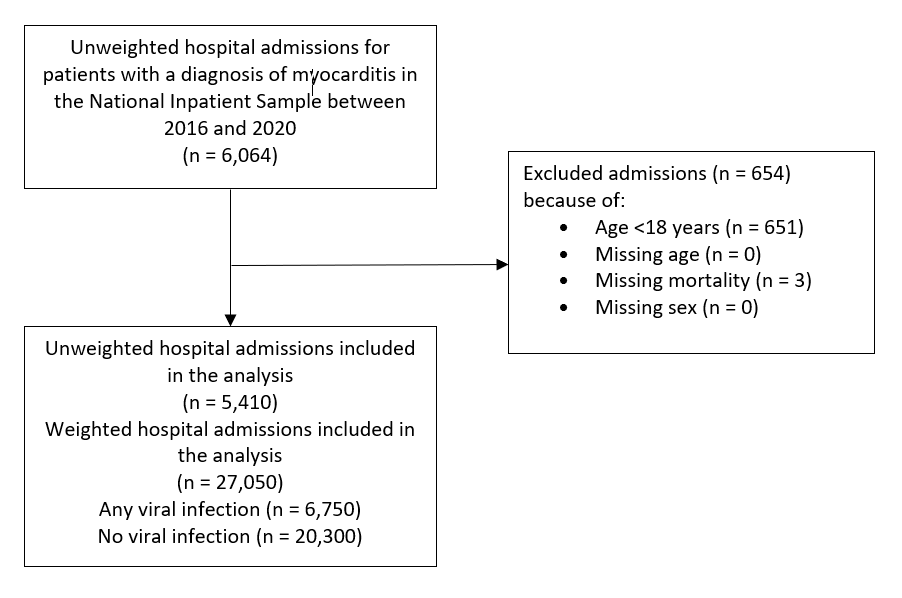
**

**Supplementary Table 1. Codes for data analysis and their source.**

| **Variable** | **Source** | **ICD-10 code** |
| --- | --- | --- |
| Influenza | I10_DX1/40 | J09*, J10*, J11* |
| Viral pneumonia | I10_DX1/40 | J12* |
| Viral gastroenteritis | I10_DX1/40 | A08* |
| Viral meningitis/encephalitis | I10_DX1/40 | A83*, A84*, A85*, A86*, A87*s |
| Herpes simplex infection | I10_DX1/40 | B00* |
| Herpes zoster infection | I10_DX1/40 | B02* |
| Acute viral hepatitis | I10_DX1/40 | B15*, B16*, B17* |
| Chronic viral hepatitis | I10_DX1/40 | B18* |
| Human immunodeficiency virus | I10_DX1/40 | B20* |
| Cytomegalovirus | I10_DX1/40 | B25* |
| Parvovirus | I10_DX1/40 | B97.6 |
| Adenovirus | I10_DX1/40 | B97.0 |
| Enterovirus | I10_DX1/40 | B97.1 |
| Respiratory syncytial virus | I10_DX1/40 | B97.4 |
| Infectious mononucleosis | I10_DX1/40 | B27* |
| Viral conjunctivitis | I10_DX1/40 | B30* |
| Any virus | - | Composite of all viral infections |
| Myocarditis | I10_DX1/40 | I40*, I41* |
| Smoking (Tobacco use) | I10_DX1/40 | Z72.0 |
| Alcohol misuse | I10_DX1/40 | F10.1 |
| Obesity | I10_DX1/40 | E66.0, E66.1, E66.2, E66.8, E66.9 |
| Systemic arterial hypertension | I10_DX1/40 | I10*, I11*, I12*, I13*, I15*, I16* |
| Hyperlipidemia | I10_DX1/40 | E78.0*, E78.1, E78.2, E78.3, E78.4*, E78.5 |
| Diabetes mellitus | I10_DX1/40 | E08*, E09*, E10*, E11*, E13* |
| Previous myocardial infarction | I10_DX1/40 | I25.2 |
| Atrial fibrillation or flutter | I10_DX1/40 | I48* |
| Valvular heart disease | I10_DX1/40 | I34*, I35*, I36*, I37* |
| Infective endocarditis | I10_DX1/40 | I33*, I38*, I39* |
| Previous stroke | I10_DX1/40 | Z86.73, I69* |
| Peripheral vascular disease | I10_DX1/40 | I73* |
| Chronic kidney disease | I10_DX1/40 | N18* |
| Liver failure | I10_DX1/40 | K72* |
| Chronic lung disease | I10_DX1/40 | J40* – J47* |
| Cancer | I10_DX1/40 | C00* – C96* |
| Dementia | I10_DX1/40 | F01*, F02*, F03*, G30*, G31* |
| Immunodeficiency | I10_DX1/40 | D80*, D81*, D82*, D83*, D84* |
| Heart failure | I10_DX1/40 | I50.21, I50.31, I50.41, I50.811  I50.22, I50.32, I50.42, I50.812  I50.23, I50.33, I50.43, I50.813  I50.1, I50.20, I50.30, I50.40, I50.810, I50.814, I50.82, I50.83, I50.84, I50.89, I50.9 |
| Sepsis | I10_DX1/40 | A41* |
| Pericarditis | I10_DX1/40 | I30* |
| Acute myocardial infarction | I10_DX1/40 | I21* |
| Endomyocardial biopsy | I10_PR1/25 | O2BL0ZX, 02BL3ZX, 02BL4ZX, 02BK0ZX, 02BK3ZX, 02BK4ZX, 02BM0ZX |
| Shock | I10_DX1/40 | R57* |
| Respiratory failure or arrest | I10_DX1/40 | J96*, R09.2 |
| Dependence on ventilator | I10_DX1/40 | Z99.11 |
| Intubation | I10_PR1/25 | OBH17EZ |
| Age | NIS Core | - |
| Sex | NIS Core | - |
| Month of admission | NIS Core | - |
| Weekend admission | NIS Core | - |
| Discharge weight | NIS Core | - |
| Discharge disposition | NIS Core | - |
| Elective admission | NIS Core | - |
| Length of stay | NIS Core | - |
| Primary expected payer | NIS Core | - |
| Race | NIS Core | - |
| Year | NIS Core | - |
| ZIP income quartile | NIS Core | - |
| Death | NIS Core | - |
| Hospital bed size | NIS Hospital | - |

**Supplementary Table 2. Co-diagnoses of viral infections among patients with a diagnosis of viral pneumonia.**

| **Diagnosis** | **Prevalence in patients with viral pneumonia** |
| --- | --- |
| COVID-19 | 80.7% |
| Influenza | 2.0% |
| Acute hepatitis | 1.2% |
| Viral gastroenteritis | 0.9% |
| Enterovirus | 0.9% |
| Herpes simplex infection | 0.6% |
| Respiratory syncytial virus | 0.4% |
| Cytomegalovirus | 0.3% |
| Herpes zoster infection | 0.3% |
| Chronic hepatitis | 0.3% |
| Viral meningitis/encephalitis | 0.2% |
| Adenovirus | 0.2% |
| Human immunodeficiency virus | 0.2% |
| Viral conjunctivitis | 0% |
| Infectious mononucleosis | 0% |
| Parvovirus | 0% |
